# Conversion disorder with aphonia in 12 years old male patient: A case report

**DOI:** 10.1016/j.ijscr.2021.106135

**Published:** 2021-06-25

**Authors:** Hasan Ibrahim Al-Balas, Mohammad Abuhalaweh, Haneen Bany Melhem, Hamzeh Al-Balas

**Affiliations:** a Faculty of Medicine, Yarmouk University, Irbid, Jordan; b Ministry of Health, Jordan; c Department of General and Special Surgery, Faculty of Medicine, Hashemite University, Zarqa, Jordan

**Keywords:** Aphonia, Conversion disorder, Vocal cords

## Abstract

**Introduction and importance:**

Conversion aphonia is a rare disease characterized by total loss of voice. It is more commonly reported in females than males, and its diagnosis is based on a comprehensive patient's clinical history, and assessment of vocal cords and other laryngeal structures using Fiberoptic laryngoscopy. Early diagnosis and phonation have a significant role in the treatment.

**Case presentation:**

A 12-year-old medically free male patient with unremarkable medical history and normal physical, social, cognitive and emotional developmental milestones presented to the otorhinolaryngology clinic with a sudden loss of voice for a duration of 3 days with absence of other associated symptoms. A comprehensive clinical history and general examination were within normal limits, and fiberoptic laryngoscopy revealed a normal-looking laryngeal structure with intact bilateral mobile vocal cords. A diagnosis of psychogenic aphonia was the most likely cause, so speech therapy concurrent with psychotherapy was the mainstay of treatment. Improvement of the patient condition noticed and he restored his speech 4 weeks after initiation of his treatment.

**Clinical discussion:**

Psychogenic Aphonia is rare disorder with female predominance and younger age onset when it is compared to males. It is also known as functional neurological symptom disorder (FND) as it is not explained by underlying medical or neurological factors. It is often preceded by psychological trauma or stressors. Diagnosis of Psychogenic Aphonia is challenging and it is often missed and delayed. Accordingly, the delay in diagnosis may significantly affect the ultimate outcome for affected patients. Speech therapy concurrent with psychotherapy represents the mainstay of treatment.

**Conclusion:**

Being rare disorder, early recognition and diagnosis of conversion disorder with aphonia is crucial. Applying diagnostic criteria which is introduced by American Psychiatric Association in The Diagnostic and Statistical Manual of Mental Disorders (DSM) facilitate the diagnosis. Multidisciplinary approach in management of affected patients ensures better outcome.

## Highlights

• Psychogenic Aphonia is rare condition that is more prevalent among young females.
• Psychogenic aphonia is commonly preceded by a stressful life event and it is more common in patients with history of abuse.
• A previous episode of aphonia and recent upper respiratory tract infection could be a predisposing factor for this condition.
• Early diagnosis and phonation have a significant role in the treatment.

## 1 Introduction

Conversion disorder, also known as functional neurological symptom disorder (FND), is defined as functional sensory or motor symptom or group of symptoms that could not be explained by underlying medical or neurological condition. The term conversion disorder was firstly introduced by Sigmund Freud, who hypothesized that these symptoms which couldn't be explained by organic causes reflect an unconscious conflict [1].

Despite the unclear cause of these symptoms, patients are not feigning these symptoms [2]. However; conversion disorder is often preceded by adverse life events, trauma, or stressors. It is worth mentioning that many of those patients have history of childhood abuse, namely emotional or sexual. Moreover, those patients are more likely to have depression, anxiety, or personality disorders [3].

Patients with conversion disorder can present with a variety of symptoms such as blindness, paralysis, dystonia, psychogenic non-epileptic seizures (PNES), anesthesia, swallowing difficulties, motor tics, difficulty walking, hallucinations, aphonia, anesthesia, and dementia [2].

As mentioned before, conversion disorder is considered a functional disorder and this makes it difficult to diagnose. So, diagnostic criteria for conversion disorder were introduced by the American Psychiatric Association in The Diagnostic and Statistical Manual of Mental Disorders (*DSM*) [9].

According to these criteria; (i) The patient has at least one symptom of altered voluntary motor or sensory function. (ii) Clinical findings provide evidence of incompatibility between the symptom and recognized neurological or medical conditions. (iii) The symptom or deficit is not better explained by another medical or mental disorder. (iv) The symptom or deficit causes clinically significant distress or impairment in social, occupational, or other important areas of functioning or warrants medical evaluation.

In this paper, we are focusing on functional aphonia that describes a patient who presented with a whispered voice on phonation without having a visible mucosal disease in the larynx [4]. This case is reported in concordance with the SCARE 2020 criteria [10].

## 2 Case report

A 12-years-old male student with unremarkable medical and surgical history was referred to our otorhinolaryngology clinic on July 2020 at Princess Basma Hospital in Jordan. He presented with his parents complaining from a sudden onset aphonia for 3 days duration. According to his parents, the patient has normal physical, social, cognitive and emotional developmental milestones. There was no history of upper respiratory tract infection, laryngeal trauma, fever, or cough. Patient is not on any medical treatment with unremarkable family history. They insisted that he suddenly had this problem following diving in swimming pool.

**Fig. 1:**
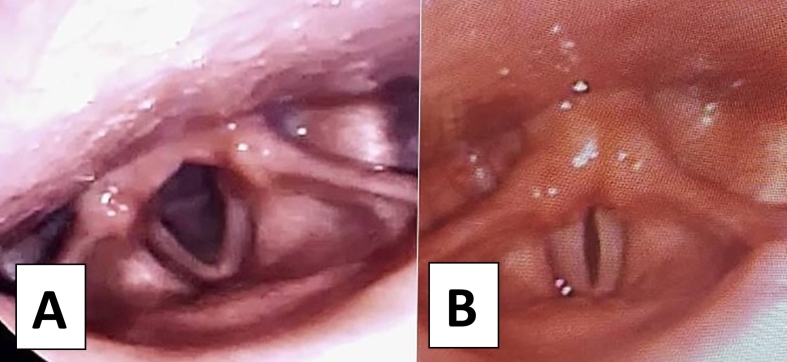
Laryngoscopy. (A) Vocal folds in abduction (breath), (B) vocal folds in abduction (phonation).

Clinically, the patient was afebrile with stable vital signs. He was conscious, alert, and following commands precisely, and examination of his ears, nose and throat were unremarkable. A flexible fiberoptic laryngoscopy under local anesthesia was performed by an otorhinolaryngology consultant. The larynx showed a mobile bilateral vocal cord with complete Aphonia (Fig. 1). There were no polyps, nodules, or compressive masses over the vocal cords. Phycological and neurological consultations were requested.

Based on overall assessment, patient was diagnosed with psychogenic aphonia, and his condition was discussed with his parents, speech therapist and psychologist. Following 3 weeks of intensive speech therapy sessions, patient showed significant improvement in his voice, and his treatment was continued for a total of 5 weeks. Patient was followed clinically for 6 months with no recurrence of previous symptoms.

## 3 Discussion

Psychogenic Aphonia is rare disorder with a point prevalence of 0.4%. Females are 8 time more likely to develop it than males [5]. Moreover, female patients who are more prevalent seems to have onset at a younger age when it is compared to males. In one study about 30 patients with psychogenic aphonia who were assessed in the department of Logopedics and Phoniatrics, University Hospital, Lund, Sweden, during the period January 1986 through October 1992, majority of patients were females (*n* = 25). Male patients in this group were older; ranging from 46 to 64 years old [6]. However, among 14 patients, three of them had suffered a previous episode of aphonia, and 11 patients had upper respiratory tract infections either before or at the onset of Aphonia.

Diagnosis of Psychogenic Aphonia is challenging, which resulted in unnecessary instruments based on over-diagnosis and significant delay in the diagnosis. In one study published by Harris et al., a total of 14 female patients with psychogenic Aphonia who were first diagnosed at the ENT department of the Saarland University Clinic were reported. The mean time interval between the onset of symptoms and the diagnosis was 9 weeks [4]. Some patients required approximately 32 weeks to be diagnosed [7]. In comparison, only 2 weeks were required to reach a diagnosis for our patient.

The mainstay treatment of psychogenic Aphonia is speech therapy in concordance with psychotherapy. Among the 14 patients reported by Harris et al., all female patients were cured by only speech therapy [4]. Moreover, in a longitudinal study on 500 patients over 32 years (1972–2004), 82% (*n* = 410) showed voice return on the first day of voice therapy [8]. On the other hand, there is a concern regarding patients who are not induced to produce vocalization immediately to develop a fixation on their Aphonia, so this makes early diagnosis and vocalization significantly affecting the outcome [5].

## 4 Conclusion

Psychogenic Aphonia is rare condition that is more prevalent among young females and usually preceded by stressful life event. A previous episode of aphonia and recent upper respiratory tract infection could be predisposing factors for this condition. Early diagnosis and phonation have a significant role in the treatment.

## Ethics approval

No institutional ethics approval is required for publishing case reports.

## Funding

The author(s) received no financial support for the research, authorship and/or publication of this article.

## Informed consent

Written informed consent was obtained from the patient for publication of this case report and accompanying images. A copy of the written consent is available for review by the Editor-in-Chief of this journal on request.

## Provenance and peer review

Not commissioned, externally peer-reviewed.

## Ethical approval

No ethical approval required.

## Consent

Written informed consent was obtained from the patient father for publication of this case report and accompanying images.

## Author contribution

**Hasan Al-Balas**: Conceptualization, Methodology, Writing - original draft, Supervision.

**Mohammad Abuhalaweh**: Writing - original draft, Writing - review & editing.

**Haneen Bany Melhem**: Conceptualization, Data curation.

**Hamzeh Al-Balas**: Data curation, Writing - review & editing.

## Registration of research studies

Not applicable.

### Guarantor

Dr. Hasan Al-Balas.

Assistant professor of Otorhinolaryngology

Faculty of Medicine

Yarmouk University, Irbid, Jordan

hasanalbalas@yahoo.com

## Declaration of competing interest

No conflict of interest between authors.
